# Challenges in predicting the evolutionary maintenance of a phage transgene

**DOI:** 10.1186/1754-1611-8-21

**Published:** 2014-08-01

**Authors:** Matthew Schmerer, Ian J Molineux, Dilara Ally, Jabus Tyerman, Nicole Cecchini, James J Bull

**Affiliations:** 1 Center for Computational Biology and Bioinformatics, The University of Texas at Austin, Austin, TX, USA; 2 Department of Molecular Biosciences, The University of Texas at Austin, Austin, TX, USA; 3 Institute for Cellular and Molecular Biology, The University of Texas at Austin, Austin, TX, USA; 4 Bayer Crop Science - Biologics, 1540 Drew Ave, Unit 170, Davis, CA, USA; 5 Total New Energies USA Inc., 5858 Horton Street, Suite 253, Emeryville, CA, USA; 6 Department of Integrative Biology, The University of Texas at Austin, Austin, TX, USA

**Keywords:** Phage, Evolution, Biofilm, Tragedy of the commons, Synthetic biology

## Abstract

**Background:**

In prior work, a phage engineered with a biofilm-degrading enzyme (dispersin B) cleared artificial, short-term biofilms more fully than the phage lacking the enzyme. An unresolved question is whether the transgene will be lost or maintained during phage growth – its loss would limit the utility of the engineering. Broadly supported evolutionary theory suggests that transgenes will be lost through a ‘tragedy of the commons’ mechanism unless the ecology of growth in biofilms meets specific requirements. We test that theory here.

**Results:**

Functional properties of the transgenic phage were identified. Consistent with the previous study, the dispersin phage was superior to unmodified phage at clearing short term biofilms grown in broth, shown here to be an effect attributable to free enzyme. However, the dispersin phage was only marginally better than control phages on short term biofilms in minimal media and was no better than control phages in clearing long term biofilms. There was little empirical support for the tragedy of the commons framework despite a strong theoretical foundation for its supposed relevance. The framework requires that the transgene imposes an intrinsic cost, yet the transgene was intrinsically neutral or beneficial when expressed from one part of the phage genome. Expressed from a different part of the genome, the transgene did behave as if intrinsically costly, but its maintenance did not benefit from spatially structured growth per se – violating the tragedy framework.

**Conclusions:**

Overall, the transgene was beneficial under many conditions, but no insight to its maintenance was attributable to the established evolutionary framework. The failure likely resides in system details that would be used to parameterize the models. Our study cautions against naive applications of evolutionary theory to synthetic biology, even qualitatively.

## Background

Biofilms are persistent bacterial structures that arise in many medical and industrial applications. They are usually undesirable and difficult to control because they are recalcitrant to treatments that eliminate planktonic bacteria [1,2]. Bacterial viruses – phages – seem to offer a promising technology for biofilm control because they are predators of bacteria and should in theory amplify and persist at the site of biofilms as long as their prey persists. Yet although phage kill bacteria, they are not necessarily endowed with the means to propagate themselves through or degrade a biofilm’s extracellular matrix. Without the ability to penetrate a biofilm, phage may merely graze on planktonic cells exuded by a biofilm without degrading or effecting a long term reduction in bacterial numbers [3].

Lu and Collins [4] proposed using engineered phages to enhance biofilm degradation. Their specific application was the addition of a gene encoding the bacterial enzyme dispersin B to the lytic coliphage T7. Dispersin degrades poly-N-acetyl-glucosamine (PNAG), an essential component of *E. coli* biofilms. The feasibility of their method in short term biofilms was demonstrated with the engineered phage providing better clearing and cell killing than did various control phages lacking the enzyme.

The purpose of the present study is to explore the evolution of this engineered phage – whether it maintains the transgene as the phage grows on biofilms. Dispersin performs its biofilm-degrading function only after an initial cell has been infected, lysed and released the enzyme into the environment. Because the enzyme performs its function outside the cell, the transgene may be lost through an evolutionary ‘tragedy of the commons’ in which the gene benefits not only its own genome but the genomes of unrelated phages lacking the gene. A considerable body of theory and empirical work has supported the tragedy of the commons framework at the organismal level [5-12], but it is not known whether and how that framework can be applied to transgenic phages. If the dispersin transgene is evolutionarily stable, it will help alleviate concerns for other engineered phages and provide impetus for their use. If unstable, can a better transgene design extend its longevity? The hope is that the evolutionary theory can provide broad insight to the fate and design of engineered phages across many contexts and system-specific details.

## The theory

### Basics

The addition of a gene to a genome will often have deleterious effects – a cost, *c*. We conjecture that a cost is nearly universal for an arbitrary transgene but that the bases of that cost lie in intricate molecular details of the phage life cycle, so the magnitude of the cost will be sensitive to details and be quantitatively unpredictable. A transgene may also provide a benefit (*b*), and the basis of this benefit will often be known or inferred in advance, as it is the motivation for the engineer. It is straightforward to appreciate that a transgene will be favored only if the benefit outweighs the cost: *b*>*c*, but understanding how these costs and benefits interact is not always straightforward. For example, in our case, any cost likely operates during the phage intracellular life cycle, whereas benefit comes from modifying the external environment in which the phage grows. The benefit thus varies with environment, but the specific nature of this benefit presents yet an additional problem.

We study a phage genome encoded to produce a transgenic enzyme, dispersin. A transgenic protein of this type is produced within the infected cell, but the expected benefit is to the phage progeny after the bacterium is lysed. After lysis, the enzyme diffuses extracellularly where it can exert its effect on the biofilm exopolysaccharide, releasing bacteria from the biofilm. The benefit is likely one of exposing bacteria to phage that would otherwise be protected by the biofilm matrix. Thus this benefit may well accrue to phages other than the ones encoding the transgene – any phage in the local environment should benefit from the cells exposed by dispersin. Consequently, the transgenic phage may be subject to an evolutionary ‘tragedy of the commons’ in which it experiences a net benefit of the transgene, but other, non-producing phages experience an even greater benefit because their genomes do not suffer the cost. The tragedy is that, even though the transgene is beneficial, phages lacking it are evolutionarily superior and the transgene is outcompeted to extinction.

The foundation for this problem thus rests on three premises. First, there is likely an intrinsic cost to the transgene of unknown magnitude and perhaps little more than a generic consequence of disrupting a wild-type genome (see Discussion for support). Second, the transgene provides a benefit, one that applies only in some environments. There may be many contexts in which the cost exceeds the benefit, but as the benefit depends on environment, there may also be environments in which the benefit exceeds the cost. Third, the specific nature of the benefit is transferable to other phages, leading to the possible evolutionary demise of the transgene even when its benefit exceeds the cost. Avoiding this sharing of benefits is key to evolutionary success of the transgenic phage, as will be expanded upon below.

### A mathematical model

The evolutionary process described above is unintuitive, so we offer a mathematical model. Following several precedents [13,14], the model uses differential equations to describe the dynamics of phage and bacteria in an environment with continuous flow, such as a chemostat, equivalent to a constant death rate. The continuous flow is represented as a constant washout/death rate of all variables. The model assumes mass action, which is most prone to a tragedy of the commons. A more accurate model of the process would include spatial structure (e.g., use partial differential equations), but the main points can be illustrated with the present model without undue emphasis on the mathematics.

The full model (1) has 8 equations that accommodate two bacterial populations and two phage types. One bacterial population exists in a ‘free’ state and is infected by both types of phages. The other bacteria exist in a refuge that fully escapes infection; the refuge population can be considered a physical region free of phages, or it can equally be regarded as planktonic cells whose surface properties protect them from infection. However, refuge bacteria are released at a low rate into the free state, at which point they are susceptible to infection.

The two phage types consist of (a) a genetically modified (GM) phage and (b) a ‘wild’ phage. The GM phage produces a diffusible enzyme whose concentration in the environment (*E*) increases the release rate of refuge bacteria into the free population; the wild phage does not produce the enzyme. The two phages have identical fitness components except for burst sizes (Table 1). In some parameterizations, the GM phage has a lower burst size than the wild phage (17 versus 20), in others, the GM phage has the higher burst size (20 versus 17), in yet others, each phage has a burst size of 20. All other fitness components are the same between both phages.

**Table 1:** Model variables and parameters

**Notation**	**Description**	**Values**
Variables		
*B*	Density of free bacteria	
*R*	Density of refuge bacteria	
*P*_*G*_	Density of GM phage	
*P*_*W*_	Density of wild phage	
*E*	Density of enzyme produced by GM phage that increases the release rate of refuge bacteria into the free state rate of both phages	
*I*_*G*_	Density of bacteria infected with GM phage (before lysis)	
*I*_*W*_	Density of bacteria infected with wild phage (before lysis)	
Functions of variables		
h(E)	Rate at which refuge bacteria move to free state	
Parameters		
*k*	Adsorption rate constant	10^-9^(mL/min)
*w*	Washout/death rate	0.05
*b*_*G*_	Burst size of GM phage	17, 20
*b*_*W*_	Burst size of wild phage	17, 20
*L*	Lysis time (min)	10
*v*	Maximum bacterial growth rate	0.1
*Z*	Enzyme production per lysis	0.1
*C*	Carrying capacity of environment	5 ×10^9^

Parameters and variables are defined in Table 1; bacterial densities are denoted *B* and *R* (free and refuge, respective), phage densities as *P*_*G*_ and *P*_*W*_. Bacterial growth in both the free and refuge populations obeys a logistic function with carrying capacity *C* that applies to the total density of bacteria, *B*+*R*. Lysis is modeled as a delay function *L* minutes after infection, hence a subscript *L* (e.g., *B*_*L*_, $P_{W_{L}}$) indicates the value of the variable *L* minutes in the past. The baseline rate at which refuge bacteria are released into the free state is 0.01 (per min), increasing up to 0.2 with *E* according to the function *h*(*E*).

In the equations that define this model, a superior dot (*˙*) indicates a derivative with respect to time:

(1) $$\;\begin{array}{l} \;\dot{B}\;=\;v\left(1-\frac{B+R}{C}\right)B-{\text{kP}}_{G}B\;-kP_{W}B\;-\;\text{wB}+\;\text{Rh}(E)\\ \;\dot{R}\;=\;v\left(1-\frac{B+R}{C}\right)R\;-\;\text{Rh}(E)\\ {\dot{P}}_{G}\;=b_{G}\;kP_{G_{L}}B_{L}\left[e^{-\text{wL}}\right]-\;kP_{G}\left(B\;+\;I_{G}\;+\;I_{W}\right)-{\text{wP}}_{G}\\ {\dot{P}}_{W}\;=b_{W}\;kP_{W_{L}}B_{L}\left[e^{-\text{wL}}\right]-\;kP_{W}\left(B\;+\;I_{G}\;+\;I_{W}\right)-wP_{W}\\ \;{İ}_{G}\;=-kP_{G_{L}}B_{L}\left[e^{-\text{wL}}\right]+\;kP_{G}B-wI_{G}\\ \;{İ}_{W}\;=-kP_{W_{L}}B_{L}\left[e^{-\text{wL}}\right]+\;kP_{W}B-wI_{W}\\ \;Ė\;=\text{Zk}P_{G_{L}}B_{L}\left[e^{-\text{wL}}\right]\;-\;\text{wE}\\ h(E)\;=\;\frac{0.2}{1+19\left(0.999997^{E}\right)}\;\;\;\;\;. \end{array}$$

**Figure 1:**
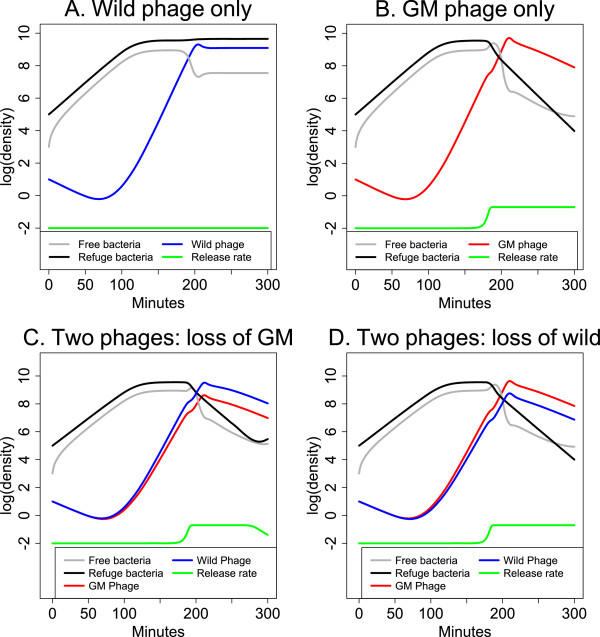
**Short term phage-bacterial dynamics and a tragedy of the commons between two phage types.** Free bacterial densities are in gray, refuge bacterial densities in black, genetically modified (GM) phage densities in red, and wild phage densities in blue. The rate at which refuge bacteria are released into the free state is in green, affected by enzyme density (not shown). Panels **(A)** and **(B)** represent cultures infected with single phage types. **(A)**: The wild phage type has little effect on bacterial densities because it does not cause release of refuge bacteria; it maintains high levels by feeding on free bacteria released from the refuge. **(B)**: The GM phage produces an enzyme that releases refuge bacteria. At high phage densities, refuge bacteria are released and killed rapidly, bacterial densities drop, and phage densities follow. **(C)**: A tragedy of the commons. Both phages are present, and in the short term, the overall phage and bacterial dynamics follow a similar pattern as when only the GM phage is present, with refuge bacteria depleted (as in B). The wild phage has a slightly higher burst than the GM phage (20 versus 17); although both phages start out equally abundant, the GM phage declines and is eventually lost if the run is continued further, the dynamics returning to the pattern in (A). Loss of the GM phage is the ‘tragedy,’ since the GM phage releases refuge bacteria. **(D)**: As in (C), but the wild phage is given the lower burst size (17 versus 20) and it is progressively lost. There is no tragedy because GM phage is maintained. When the GM phage is lost (in C), there is an earlier recovery of bacterial densities and earlier return of the release rate to baseline than in (D). The scale of the vertical axis is log _10_.

Representative short term dynamics are illustrated in Figure 1 and do not require understanding the equations. The top two panels show outcomes for environments with single phages, each with identical parameters except for enzyme production. In each, phage densities start out low and increase in response to the abundance of hosts. In (A), the wild phage cannot infect the refuge population because they cannot free it, so phage and bacteria are both maintained at high density – the refuge population continually generates free bacteria on which the wild phage preys. This equilibrium continues indefinitely. In (B), the GM phage frees refuge bacteria and exposes them to attack; the total bacterial density declines several logs once the phage reach high density, and because hosts are no longer abundant, phage densities then decline as well. If continued longer, the bacteria would rebound, and the phage would again rise to suppress their numbers; this longer time frame is not necessarily relevant to phage treatment applications. Because the GM phage is able to destroy the refuge bacteria, its numbers briefly spike above those of the wild phage (in A), but then the paucity of hosts causes GM phage densities to fall below those of the wild phage. The green curves show the (log _10_) rate at which refuge bacteria are being released into the free population.

The lower two panels illustrate cultures with both phages together; in each panel, one phage is given a lower burst size than the other (17 for one, 20 for the other). Both lower panels show the same general pattern seen in (B) of phages increasing in abundance initially, eventually overwhelming both bacterial populations, and then declining for lack of abundant hosts. However, of specific interest in (C) and (D) are changes in the *relative* abundances of the two phages (the relative heights of the two phage curves) and the effects of those relative changes on the bacterial dynamics. The two phages start out at 50% frequency in both panels. In (C), the GM phage has the smaller burst and it is progressively lost (dropping to 9% frequency by the end). In (D), the wild phage has the smaller burst, and it is progressively lost (dropping to 10% frequency by the end).

Although the GM phage is responsible for the major impact on bacterial densities (as seen by comparing (A to B), it is progressively lost when the wild phage has the larger burst (in C), which is the tragedy of the commons. The effect of wild phage dominance in (C) is already evident as a reduced impact on and faster rebound of bacterial densities as well as an earlier decline in the release rate of refuge bacteria (compare C to D). If the run in (C) was continued, the system would return to the equilibrium in (A), with a high density of refuge bacteria and only the wild phage present. If the run in (D) was continued, the wild phage would be lost, and both the bacteria and GM phage would cycle between high and low frequencies.These figures are representative of additional runs (not shown). The details of the dynamics vary (e.g., oscillations dominate some parameter values, densities equilibrate for others), but the GM phage is always progressively lost when it has the lower burst size. Thus the benefit it provides by exposing refuge bacteria does not contribute to its maintenance when both phages are present. It may seem that a tragedy of commons is not relevant when only a single phage type is introduced to a system (e.g., Figure 1B). However, mutations that delete the transgene often quickly arise and convert the population to a mix of types with and without the transgene.

### Can the tragedy be avoided?

A widely acknowledged principle for avoiding an evolutionary tragedy of the commons is to impose spatially structured growth [5,7,11,15]. Spatial structure enables an individual and its descendants to be ‘selfish’ by restricting diffusion of its products just to immediate relatives. The spatial structure must be refreshed often enough (e.g., by dispersal and reestablishment) that selfish mutants do not have time to invade. In theory, an engineered phage introduced into a biofilm might avoid the tragedy if there was adequate structured growth. Some observations of phages in biofilms support structured growth [16], but many dynamical details are unknown, and there may also be a substantial level of phage ‘grazing’ on planktonic cells produced by biofilms [3].

There is, however, a second layer to the tragedy of the commons that applies to phages: spatially structured growth can work against phages engineered to kill bacteria efficiently and rapidly. In the absence of spatial structure, when phages are mixed in direct competition, phages with higher growth rate have the advantage because they are the fastest to use up all the hosts [10]. In contrast, with spatial structure, phages with low-intermediate growth rates have the advantage because they use their hosts more ‘prudently’ by allowing host numbers to reach high levels before exploiting them. In the long run, those high bacterial densities yield high levels of phage progeny production – higher progeny levels than achieved by phages that kill quickly. Viewed in this light, the advantage of rapid phage growth when phages compete directly, in the absence of spatial structure, is itself a tragedy of the commons [10].The benefit to the wild phage of avoiding direct competition with the GM phage can be seen by comparing phage densities in Figure 1A and B. When grown by itself, the GM phage quickly exhausts its hosts and thus realizes only a brief numerical superiority over the wild phage, but outside this window, the wild phage produces more progeny than the GM phage. If spatially structured growth operates so that there is a limited abundance of hosts available at each focus of infection, there may be only a brief window of time in which the GM phage produces more total offspring than the wild phage because it runs out of hosts.

Ironically, therefore, there are two layers to the tragedy of the commons in this problem, and they work against the GM phage both with and without spatial structure. The intrinsic high growth rate of the GM phage would give it the advantage when in direct competition with the wild phage (in the absence of spatial structure) except that it shares its enzyme and thus also boosts the wild phage growth rate - a tragedy that works against the GM phage. Yet the fact that the faster-growing phage wins the direct competition is itself a tragedy, a tragedy that would work in favor of the GM phage if it did not share enzyme. The first tragedy trumps the second, however. Yet when the phages grow with spatial structure, both tragedies are avoided and the wild phage can produce more progeny in the long run because it does not exhaust its hosts quickly.

It is of course possible that spatially structured growth of the phages would conspire to create just the right combination of dynamics to give the advantage to the GM phage. However, even if this were serendipitously achieved in the initial conditions, a further complication is that the degree of spatial structured growth may itself change and decay during phage growth. As phage densities increase and phages diffuse throughout the biofilm, there may be progressively fewer infection foci founded by single phages.

In sum, we can merely conclude at this juncture that the outcome of spatially structured competition is sensitive to subtle and likely unmeasurable details in the growth dynamics. At best, there may be a temporary phase in which the GM phage can avoid a tragedy of the commons, but we are limited to conjecture as to whether a biofilm ever provides the appropriate structure.

### A heuristic approach

The value of model (1) is heuristic. With 8 parameters and several functional forms, it cannot be parameterized, and its neglect of explicit spatial structure is also a limitation. There is little alternative, however, as phage dynamics embedded in a biofilm have never been precisely quantitfied. The model nonetheless guides us in a qualitative awareness of the conditions for evolutionary maintenance of a biofilm degrading enzyme: 

1 The transgene will be favored if it provides an intrinsic benefit – one that operates in the absence of a biofilm-structured environment (e.g., a larger burst size). This point is obvious, but we also consider intrinsic benefits to be rare outcomes – we can cite no precedent for a transgene that proved beneficial in the absence of a metabolic modification designed to favor the gene, such as drug resistance (see Discussion). However, even if an intrinsic cost proves to be nearly universal, it is conceivable that engineering can be used to minimize it.

2 A gene with an intrinsic cost can be favored if its biofilm degradation exposes cells to attack that would otherwise be protected. This condition is not sufficient however, as there must be spatially structured phage growth that strikes a delicate balance between avoiding enzyme sharing with unrelated phages and killing its hosts too quickly. At present, the dynamics of phage growth in biofilm environments have too many unknowns for these conditions to be predictable.

On theoretical grounds, the odds oppose the evolutionary maintenance of phages engineered to degrade biofilms.

## Results

The ultimate focus of this paper is evolutionary – the maintenance or loss of the dispersin transgene. Yet the selective basis of a transgene product resides in its phenotypic effect. The first part of Results thus addresses the phenotypic effect of dispersin-bearing phages in several environmental contexts. Phenotype assessment is then followed by evolutionary considerations.

### 1. The dispersin phenotype meets expectations

Lu and Collins [4] used a biofilm-clearing assay of intact phages to infer activity of the dispersin transgene. T7 with dispersin outperformed the parental, empty T7 vector as well as a wild-type T3 control. However, T3 substantially outperformed the empty T7 vector, raising the possibility that the dispersin transgene may have augmented biofilm clearing in ways that did not exploit its enzyme activity. Thus we first explore the nature of dispersin’s activity in clearing biofilms: (i) Is biofilm clearing robust to growth conditions? (ii) Does dispersin enzyme alone have the expected effect? Our assays used short-term biofilms broadly similar to those of Lu and Collins – overnight bacterial growth in polystyrene 24-well microtiter plates followed by phage treatment, then assayed with crystal violet (CV) staining. The bacterial strain used was a *csrA* deletion mutant of *E. coli*, which overproduces the PNAG substrate digested by dispersin; the mutant exhibits enhanced biofilm production. Untreated and unstained, these overnight biofilms were evident as thin films (less than 1 mm thick) on the bottom and sides of a well.

Various dispersin phages were tested. The specific notation for a phage is T7*insert*_*l* *o* *c* *a* *t* *i* *o* *n*_, where *insert* indicates the set of genes added to the phage genome, and the subscript _*l* *o* *c* *a* *t* *i* *o* *n*_ indicates the cloning site. ‘T7dsp’ is used generically for convenience when the specific genome has been made clear from the context.

#### 1.A. Short-term biofilms: transgenic phage superiority across various media

**Figure 2:**
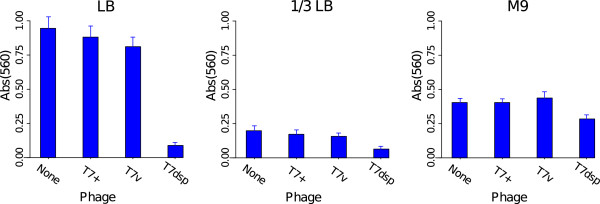
**Transgenic phage efficacy on short term biofilms.** Each panel represents a different media (full strength LB, $\frac{1}{3}$ LB, and M9 glucose) treated with the same phage stocks (T7+ is wild type T7, T7v is the empty T7 vector, and T7dsp is T7dsp+trx _10*B*_). The vertical axis is the absorbance (560 nm) of the eluent after crystal violet staining. The bars give mean absorbance with 1 std error. Across each media, there is significant heterogeneity when and only when treatment with T7dsp is included (only marginally so for M9). There is also significant heterogeneity among the T7dsp treatments across the 3 media due to the M9 glucose experiment. Lower signal indicates greater biofilm clearing.

Short-term biofilms were grown as overnight cultures at 37° in 24-well, treated polystyrene microtiter plates, then incubated with phages for 5 h, and finally assayed for clearing with crystal violet (CV, Figure 2). Three types of media were used, full strength broth (LB), dilute broth ($\frac{1}{3}$ LB), and minimal (M9 glucose). With such short-term bacterial growth, a strong effect of media on biofilm mass is expected and was in fact observed: across the 3 types of media, the untreated biofilms grown in full strength broth (LB) yielded the highest CV signal. For different phage treatments within the same media, the lowest CV signal was invariably with T7dsp; heterogeneity within each media was significant when all treatments were included but vanished when the T7dsp treatment was removed (when T7dsp was included, heterogeneity significance levels were P <0.0001 for LB, P <0.02 for $\frac{1}{3}$ LB, and P <0.04 for M9 glucose). However, the CV signal from T7dsp was significantly higher in M9 glucose media than in the other media (P <0.0001). For all 3 media, treatments with phages lacking transgenic dispersin were statistically indistinguishable from phage-free controls in the same media. These results support the main observations of Lu and Collins [4] but they further suggest that the magnitude of effect is media-dependent.

The model requires that the transgene benefit the phage. We have thus far merely conjectured from plausibility that a greater clearing ability translates into greater progeny production. However, Lu and Collins [4] provided assays that supported a link between biofilm clearance and phage amplification. The dispersin phage consistently showed greater bacterial killing than the control phage (their Figures 3 and 4) as well as a superiority in phage numbers soon after treatment (their Figure 4); the latter effect is visually small because it is plotted on a log scale. Most phage replication occurs on planktonic cells in the media, so the effect of killing biofilm cells is not expected to be easily noted under the conditions used.

**Figure 3:**
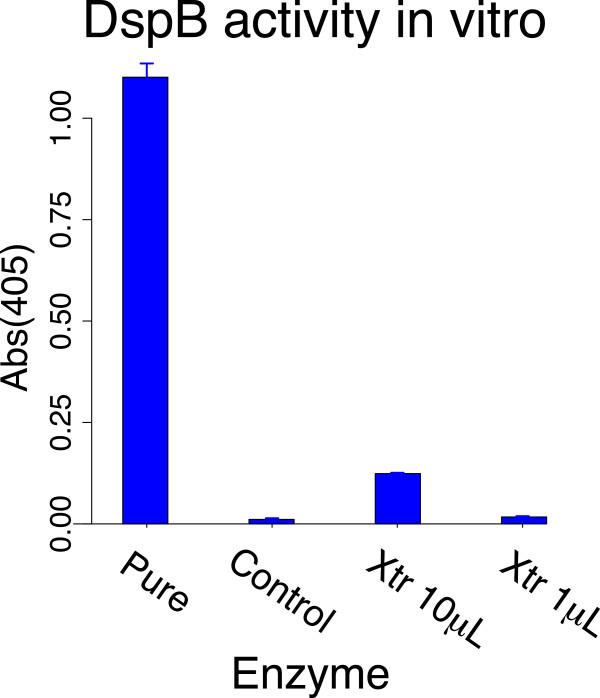
**Assays of dispersin activity using a colorimetric substrate.** Note that a high absorbance in this assay indicates high enzyme activity (in contrast to the CV assay). The Pure enzyme is a commercial preparation and shows high activity, reflecting its activity as an exoglycosidase on a substrate designed for that activity. The control is from a freeze-thaw lysate of BL21(DE3) cells carrying an empty pET15b plasmid. The bars for enzyme extract (Xtr) are from freeze-thaw lysates of BL21(DE3) cells carrying plasmid pET15b-dspB, with the volume of the extract in parentheses. Bars of 1 std. error are shown (sometimes too small to view).

**Figure 4:**
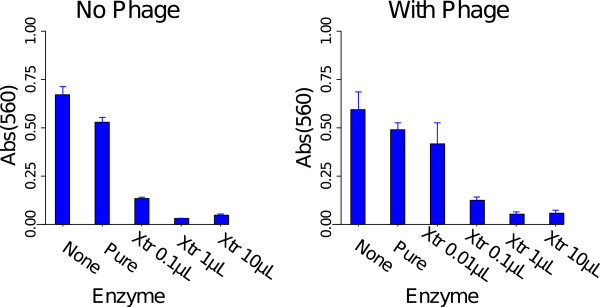
**Dispersin enzyme activity on short term biofilms without added phage (left panel) or with T7v (right panel).** After 5 hr of enzyme (with or without concurrent phage treatment), the biofilm wells were stained with crystal violet, and absorbance was measured on the eluted wash. A strong effect of enzyme is observed, and phage addition had no significant effect (no pairwise t-tests were significant). The enzyme-free control (None) is leftmost in each panel, followed by the pure, commercial enzyme (Pure), and the 3–4 rightmost histograms are for different volumes of dispersin extract (Xtr), the same as used in Figure 3. The effect of 0.01 *μ*L of extract is shown in the presence of phage primarily to demonstrate that the phage were not having an effect that was masked by enzyme. Error bars indicate 1 std error.

#### 1.B. Enzyme alone is sufficient for short-term biofilm degradation

Is the biofilm-clearing effect associated with transgenic dispersin of T7dsp due to enzymatic degradation of the biofilm matrix? Activity of the dispersin protein *per se* was measured directly with two types of assays. One used a colorimetric indicator specific to dispersin [17] and yielded a strong signal of activity with a commercially purified enzyme which is an exodepolymerase (Figure 3). In this assay, phage lysates from T7dsp-infected cells failed to produce a signal above background unless they were first concentrated with ammonium sulfate (not shown). Freeze-thaw lysates of bacteria expressing the dispersin B gene from a plasmid yielded a significant signal, approximately 10-fold less than that of the commercial enzyme (Figure 3).The second measure of dispersin activity used the short-term biofilm clearing assay (Figure 4). Enzyme was added to wells with or without T7v control phage; clearing was assessed with CV staining at 5h treatment. For enzyme from the freeze-thaw lysate used in Figure 3, a monotonic relationship was observed across dilutions, with a significant effect to at least a 100-fold lower concentration than that needed to produce a significant colorimetric signal. It is noteworthy that the commercial dispersin enzyme was totally ineffective in this assay, perhaps reflecting a different enzymatic activity than dsp B on PNAG. There was no significantly greater clearing of biofilms in which enzyme alone was added versus enzyme plus T7v.

### 2. Long-term biofilms: phage efficacy independent of dispersin

**Figure 5:**
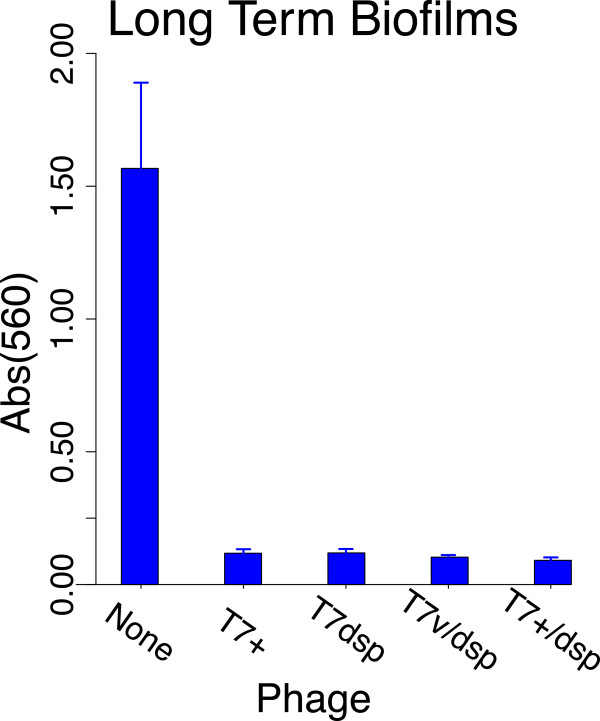
**The dispersin transgene provides no benefit over control phages to the clearing of long term biofilms.** Biofilm residue was measured with the crystal violet assay in biofilms that were grown 7 days in silicone tubing, then treated 4–5 days with phage (the leftmost bar is a no-phage control). T7+ and T7dsp (T7dsp+trx _10*B*_) represent single-phage treatments, whereas T7v/T7dsp and T7+/T7dsp are from biofilms treated with equal mixes of control and transgenic phage. No significant heterogeneity is evident across the four phage-treated biofilms. Error bars represent 1 std. error; from left to right, the number of biofilms is 6, 6, 7, 2, and 2.

Biofilms were grown 7 days in silicone tubing at 37° in $\frac{1}{3}$ LB. This combination of time and media concentration enabled a robust biofilm to develop without complications of bacterial growth into the delivery or sampling tubes. In contrast to the short term biofilms, the week-old biofilms were visibly thick (several mm) but also irregular in macroscopic structure. They were also delicate in that their structure shifted if the tubing was physically disturbed.Pure phage populations or 1:1 mixtures of T7dsp and control phages were added on day 7. After 4–5 days of phage treatment, a CV assay was performed on the contents of the tubing (Figure 5). In contrast to the superiority of dispersin phages over control phages on short-term biofilms, all phages and phage combinations cleared the biofilm mass substantially, no differences being attributable to the transgenic dispersin. To ensure that dispersin activity had not been lost during these adaptations (which could explain the lack of a difference between dispersin-containing and control phages), dispersin activity of final phage populations was tested in short term biofilm assays; these tests were conducted with eluents of two long term biofilms that had been seeded with 100% T7dsp. Clearance of short term biofilms by these eluents was compatible with maintenance of functional dispersin (not shown). Thus the equivalence of the different phages on long term biofilms was not due to loss of dispersin functionality.

These data clearly present an anomaly: dispersin is essential for clearing short term biofilms but has no significant effect on long term biofilms. It is tempting to attribute the difference to biofilm thickness or gene expression changes with age (e.g., PNAG levels may differ between short term and long term biofilms), but those features are not the only differences between short and long term biofilms. The substrate on which the two biofilms were grown also differed – silicone for the long term biofilms versus treated polystyrene for the short term biofilms. To evaluate whether substrate influenced the dispersin effect, 2 mm thick rings of sterile silicone tubing were added to wells of a polystyrene microtiter plate and a standard short term biofilm was grown; both wells and tubing were then treated with phage in the usual fashion. The polystyrene walls of the microtiter wells were specifically sensitive to T7dsp and were unaffected by control phages; yet by visual inspection the silicone rings in the same wells were equally sensitive to all phages.

The data thus suggest that the properties of a biofilm - and thus its susceptibility to phage infection - is affected by the substrate on which it is initiated. Because substrate-bound cells are only a minor component of a thick, long-term biofilm, it further suggests that the overall architecture of a biofilm is influenced by the nature of binding of that minor component.

**Figure 6:**
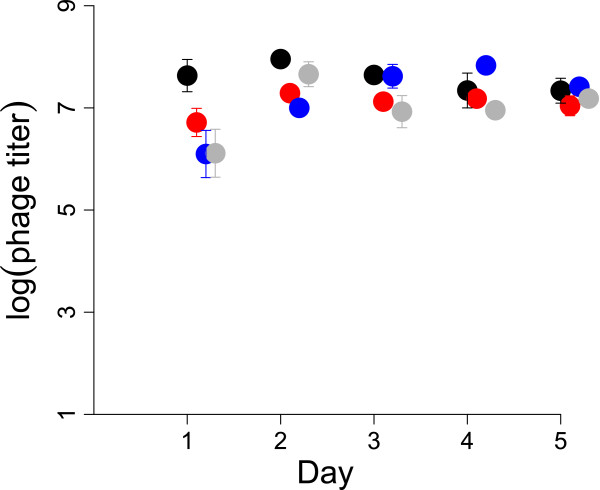
**Phage titers in the effluent of long term biofilms (the same biofilms assayed in Figure **5). The horizontal axis gives the number of days of phage treatment. Although titers one day after phage addition show some scatter, all treatments are clustered near 10^7^/mL by day 3. Red: T7dsp (T7trx+dsp _10*B*_), black: T7+, blue: an equal mixture of T7+ and T7dsp, and gray: an equal mixture of T7v and T7dsp. Error bars of ± 1 std. err. are shown. Logs are to base 10.

Phage titers were monitored daily in the outflow of the silicone tubing in which the long-term biofilm was growing. Regardless of phage identity, titers were maintained near 10^7^/mL (Figure 6). Thus despite clearing of the biofilm by the phage as revealed by CV staining, a reservoir of sensitive cells must have persisted to allow phage maintenance. The fact that phage titers were maintained at the same level across 4 days suggests minimal bacterial evolution to resist phage killing. The maintenance of phage likely reflects residual bacterial ‘wall growth’ and other refuges that the phage could not access (as noted for glass chambers in [18]), a model further supported by consideration of the biofilm flow rate: a 5 mm length of tubing experiences 90 replacement volumes of media per hour, so a population of purely suspended cells could not have maintained itself against this flow.

### 3. Evolution

The preceding results testify to the benefit of the dispersin transgene in reducing biofilms under some conditions. A question motivating our study is the evolutionary one of whether and under what conditions dispersin will be maintained. In particular, can the general framework of the tragedy of the commons be applied to understand evolution of the transgene?

#### 3.A. Transgene maintenance on long term biofilms

Our initial assays of evolution were carried out in long term biofilms and revealed that the dispersin transgene was maintained, which we interpreted as indicating spatially structured growth (Table 2). These adaptations included some biofilms inoculated with only T7dsp+trx _10*B*_ and others inoculated with a 50:50 mix of that and a control phage. The dispersin phage was lost in one biofilm and appeared to decline in two others, but it was maintained at detectable levels after 4 days of growth in 5 of 6 populations. If the dispersin gene imposed a substantial cost on the phage, maintenance of the dispersin gene in these biofilms should indicate avoidance (or reduction) of a tragedy.

**Table 2:** **Phage evolution in long term biofilms**^**1**^

**Initial phages**^**2**^	**Final dsp frequency**^**3**^	**Confidence interval**^**4**^
T7dsp	1.0	(0.94, 1)
T7dsp	1.0	(0.94, 1)
T7dsp, T7v	0.15	(0.07, 0.33)
T7dsp, T7v	0.0	(0, 0.06)
T7dsp, T7+	0.5	(0.24, 0.76)
T7dsp, T7+	0.15	(0.07, 0.33)

^1^The biofilms assayed here are a subset of those shown in Figures 5 and 6. ^2^T7dsp is T7dsp+trx _10*B*_. Where two phages were inoculated, the starting frequencies were approximately 0.5 each. ^3^After 96hr phage growth, frequencies were estimated from 8 PCR reactions per sample for each of two primer sets, each reaction comprised of 6 plaque isolates. The PCR product from one primer set indicated presence of the dispersin gene in at least one of the 6 isolates of the reaction; the product from the other set indicated its absence in at least one of the 6 isolates. Frequency was estimated as the maximum likelihood value of the corresponding multinomial term. ^4^The confidence interval was calculated as the Bayesian credible interval using a uniform prior.

#### 3.B. Dispersin cloned in *10B* is maintained on planktonic cells, indicating an intrinsic benefit

In the theory section, we suggested that transgene maintenance required either an intrinsic benefit – which seemed unlikely *a priori* – or required the right combination of spatially structured dynamics. In view of the observed maintenance of dispersin in long term biofilms (section 3.A. Transgene maintenance on long term biofilms, above), we sought to distinguish these alternatives. The straightforward way to demonstrate an intrinsic fitness benefit is to grow the phage in the absence of PNAG or the absence of spatial structure. If the gene is deleterious, it should be lost – as was observed in previous work with an endosialidase transgene in T7 [15].

Adaptations of various dispersin phages on planktonic IJ1133 (which has normal PNAG expression) revealed that the dispersin transgene was commonly maintained. Many of these outcomes were assessed qualitatively (e.g., the presence of dispersin was indicated by a positive PCR signal in the final culture), but some quantitative experiments were also conducted: (1) a 3 hr adaptation of a recombinant mixture of T7dsp+trx _10*B*_/dsp _10*B*_ on IJ1133 in LB followed by single plaque isolation revealed dispersin in all of 10 plaques tested at both the start and end of the adaptation; (2) a similar test using a T7dsp+trx _10*B*_/T7v recombinant mixture detected dispersin in 7 of 10 plaques at the start and in 5 of 10 plaques at the end. These data do not rule out a decline in the fraction of phage genomes that retained the dispersin transgene, but they indicate that any decline is at most modest.

As there is no obvious benefit to degrading extracellular PNAG in planktonic cultures of IJ1133, maintenance of the dispersin transgene evinces either an unexpected benefit to the intracellular phage life cycle or a minimal cost. This is in sharp contrast to transgenic endosialidase [15]. Nevertheless, because dispersin is maintained on planktonic cells, its maintenance in a biofilm cannot be attributed to avoidance of a tragedy of the commons (recall Figure 1D).

#### 3.C. Evolution of dispersin cloned in *3.8*: loss on liquid-grown planktonic cells, maintenance in biofilms but also in biofilm-derived planktonic cells

The genomes used in the previous assays harbored dispersin in gene *10B*, in the late or class III region of the phage. The relative costs and benefits of a transgene in different genomic locations are not yet predictable, but as the timing and levels of gene expression in T7 are affected by position in the genome [19], varying effects of insertions at different locations are to be expected. We constructed a T7dsp phage with dispersin inserted into gene *3.8*, a non-essential class II gene of no known function [19]; this phage is denoted here as T7dsp _3.8_ (as T7dsp+1.2 _3.8_/dsp _3.8_ in Table 3). One rationale for this choice was that the dispersin gene would be expressed sooner after infection in T7dsp _3.8_ than in T7dsp _10*B*_, providing a greater opportunity for it to have a deleterious effect on phage development. Indeed, a preliminary adaptation on planktonic IJ1133 revealed that the dispersin transgene was completely lost. More thorough adaptations were then conducted with a recombinant mix of that phage.

**Table 3:** Phage strains

**Notation**	**Genotype**	**Use**	**Ref**
T7+	Wild-type (Genbank V01146)	Control phage for some adaptations	[20]
T7v	T7 cloning vector, made as T7Select 415b-1 (Novagen) with the left end BclI fragment replaced by that of a phage carrying the C74 deletion (gene *0.3* is intact)	Used as backbone for gene insertions because of the cloning site and the ability to grow on hosts containing type I restriction modification (RM)	T7 _0_ in [15]
T7dsp+trx _10*B*_	T7415-C74:trxAdspB. T7v with the *E. coli trx*A gene, T7 *ϕ*10 promoter and *A. actinomycetemcomitans* CU1000 *dsp*B gene recombined in to the multiple cloning site between genes *10* and *11*	Re-engineered version of T7DspB from [4] to grow on hosts with type I RM	This study
T7dsp+trx _10*B*_/ dsp _10*B*_	T7dsp+trx _10*B*_ grown on a host with pUC57-T7dspB to remove the *trx*A gene by recombination; not clonally isolated	Used to allow evolution of dispersin free of *trx*A	This study
T7dsp+1.2 _3.8_	T7v but most of gene *3.8* replaced with T3 gene *1.2* (enabling growth on F plasmid-bearing strains) and the *dsp*B gene. Created by recombination with plasmid pMK-RQ-T7trxAdspB. The multiple cloning site in gene *10B* is unaltered from T7v.	Used to create T7-1.2+dsp _3.8_/dsp _3.8_.	This study
T7dsp+1.2 _3.8_/ dsp _3.8_	T7-1.2+dsp _3.8_ recombined with pMKRQ-T7dspB to create variation in the presence/absence of the *1.2* gene. Not purified.	Evolved in a chemostat to study evolution of the dsp gene.	This study
T7dsp _3.8_P	The initial mix of T7-1.2+dsp _3.8_/dsp _3.8_ recombined against the chemostat-evolved population of T7-1.2+dsp _3.8_/dsp _3.8_ in which the dsp gene had been lost.	Multiple recombinations separated dsp from *1.2* and introduced any mutations from a chemostat adaptation that might be beneficial in the background of the phage. This polymorphic population was used to evaluate the effect of selection on the dsp transgene in serial transfer, short term biofilms and long term biofilms.	This study

Starting with a T7dsp _3.8_ shown to have dispersin activity, a polymorphic population was created (denoted T7dsp _3.8_P) in which 26% of the phage carried dispersin [credible interval (0.18, 0.36)]. By starting with a mix of different genotypes at intermediate frequencies – as opposed to a pure type – selection quickly leads to an easily detectable change in frequency of the dispersin transgene [21]. Serial transfer of T7dsp _3.8_P for 10–12 hr (∼ 40–50 generations) at 37° on planktonic *csrA* cells in $\frac{1}{3}$ LB broth resulted in apparent loss of the dispersin gene in both of two replicates (undetected in 50 plaques of the final population from one replicate, 40 in the other). The loss of dispersin in liquid serial transfer indicates an intrinsic cost and renders this phage appropriate for testing the role of spatial structure in avoiding a tragedy of the commons. If the biofilm environment is selective for dispersin and if the spatial structure provided by a biofilm avoids a tragedy of the commons, the dispersin gene frequency should be constant or even increase in biofilms.

##### 3.C.1. Long term biofilms

**Figure 7:**
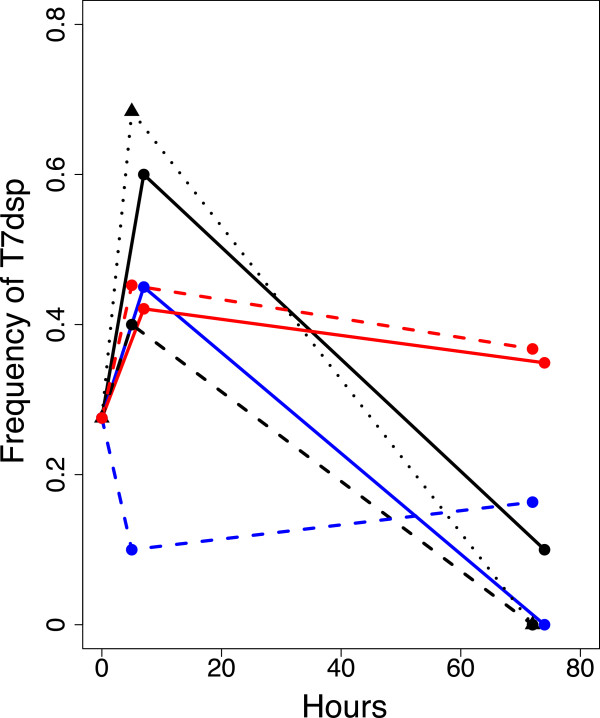
**Frequency of T7dsp during growth in long term biofilms.** The phage inoculum was T7dsp _3.8_P. Each color-texture line combination represents a different biofilm, each inoculated with the same phage stock; the frequency of T7dsp in that stock is used as the initial frequency for all 7 adaptations. The black dotted line is for cells sloughed from a 7-day biofilm and shunted into a biofilm-free tube two hours before phage addition; those cells should be almost entirely planktonic.

T7dsp _3.8_P was introduced to 7-day biofilms. In principle, long term biofilms enable phage evolution to be studied across days rather than hours. However, if a phage destroys the biofilm environment, it may destroy any spatially-structured selection for maintaining the dispersin gene. Typically, the visibly thick matrix of long term biofilms was cleared within 12–24 hr after phage addition, so sampling was conducted early (5–7 hr) and late (70+ hr). In 5 of 6 biofilms, the dispersin transgene initially rose in frequency over 5–7 hours but then declined by 72–74 hr; in the sixth biofilm, an initial decline was followed by a slight rise (Figure 7). Collectively, the dispersin transgene appears to be favored or maintained early in biofilm attack but not long term.To distinguish any benefit of structured growth from the physiological state of the bacterial host on maintenance of the dispersin transgene, evolution of dispersin was tested in cells being sloughed off a long-term biofilm. At day 7 of bacteria-only biofilm growth, the effluent from a biofilm was shunted into fresh tubing, which therefore should contain primarily planktonic cells. After 2 hr incubation, phage were added. Evolution of T7dsp shows the same pattern on the shunted cells as on the established biofilms, only more so: a sharp rise in the frequency of the transgene in the short term but loss in the long term (dotted black in Figure 7). This result suggests that the early benefit of T7dsp depends more on the physiological state of the cells than on spatial structure.

##### 3.C.2. Short term biofilms

The dispersin transgene has a dramatic effect on short term biofilms. It was thus of special interest to determine whether the dispersin transgene would also realize a major evolutionary advantage in that treatment. For reasons noted above, it was necessary to use T7dsp _3.8_P for this test, as the transgene appears to be intrinsically beneficial in T7dsp _10*B*_. To control for as many variables as possible while comparing the effect of planktonic versus biofilm-embedded cells, planktonic cells were obtained from the first rinse of an overnight biofilm well using fresh media and deposited in an empty well where no biofilm was present. (Microscopic observations on washes confirmed that cells were either single or in small clusters of 5–20 cells that should allow free phage access independent of dispersin.) The well containing the original biofilm was replenished with fresh media. Thus one well had a biofilm and the other had only planktonic cells derived from it. T7dsp _3.8_P was added to both wells, and the frequency of the dispersin transgene compared between them after 5 hr of growth.

**Figure 8:**
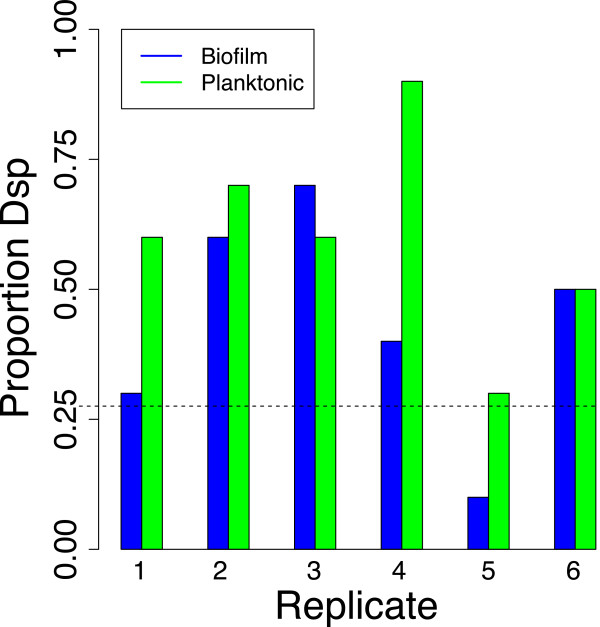
**Frequency of phage carrying a dispersin transgene after 5hr growth on cells derived from short term biofilms.** Each pair of bars represents an independent, paired replicate: a blue bar is from phage growth on a biofilm, the adjacent green bar is from growth on planktonic cells derived from that biofilm. For each bar, the frequency of dispersin is the fraction of 10 plaque isolates from the final population that carried the gene. The horizontal dotted line is the initial frequency of the dispersin transgene in the T7dsp _3.8_P stock added to the cells.

Surprisingly, there was no significant difference between the planktonic and biofilm environments (Figure 8). Equally surprising, the frequency of T7dsp increased significantly from its initial value in both environments (combining the 6 replicates and using 0.26 as the expected frequency, *χ*^2^(1)= 35.7, P <10^-4^ for the planktonic cells, = 9.2, P <0.0024 for the biofilm cells). Thus, the profound importance of dispersin in clearing short term biofilms is matched by an apparent selective advantage of the dispersin gene, but any spatial structure of the biofilm has no additional effect.

## Discussion

Organisms with engineered genomes are finding ever-increasing applications (e.g., [22-25]). Commonly, those engineered organisms must reproduce themselves to perform their function, whether in a fermenter, patient, or field. That reproduction virtually ensures their evolution, evolution that may reverse the engineering, improve it, or allow its escape. Being able to predict that evolution will have many benefits that span social impacts to corporate finances. The present study is broadly viewed as a test case of this perspective, predicting the evolution of an engineered genome. Overall, the results challenge the expectation that evolution can be predicted from general principles applied naively.

The effort here used an established transgenic phage system. Lu and Collins [4] engineered a T7 to encode the bacterial gene for dispersin, which degrades an exopolysaccharide component of biofilms (poly-N-acetyl-glucosamine or PNAG). In support of their design principle, the engineered phage cleared short-term biofilms more completely than did control phages. What was not tested was whether the phage would retain the dispersin gene and activity during growth in biofilms. This latter issue is one of evolutionary stability, and it intersects the domains of ecology and evolution, where there are well established principles that might be expected to govern the fate of engineered phages [5-12].

The crux of the evolutionary problem faced by this phage was thought to reside in a framework known as ‘tragedy of the commons’. It was assumed that dispersin would not be beneficial to the intracellular phage life cycle, but that it might benefit the phage population by degrading the biofilm matrix and thereby increasing phage access to cells; data from Lu and Collins supported this latter point. But a benefit to the phage population is not the same as a benefit to the transgenic genome: the dispersin protein is released at lysis as a free protein that diffuses and degrades the matrix in the local environment. Thus any and all phages in this local environment benefit, not just the genome that produced the enzyme. The intrinsic cost of the transgene – incurred only by the genome encoding it – then leads to its evolutionary loss when competing with phages lacking the transgene.

Well established theory suggests that the tragedy might be avoided if the phage is ‘selfish’, as by growing in a physically structured environment that limits sharing of the transgenic protein. But even this solution requires a delicate balance of dynamical properties. Based on these *a priori* considerations, evolutionary maintenance of the dispersin transgene seemed unlikely but not impossible, but that spatially structured growth was required.

The naive application of the evolutionary framework failed because one of the main assumptions failed: the transgene was not intrinsically deleterious. It is of course possible to offer reasons *post hoc* why an arbitrary transgene might have virtually no cost (e.g., restoring genome length closer to wild-type), but previous work points to nearly ubiquitous deleterious effects of engineered changes in T7 [15,26,27] (including unpublished observations on *trx*A inserts) and even bacteria [28]. The failure is thus one of the approach used here, extrapolating seemingly generic properties of transgene effects observed in other contexts to dispersin. In short, details matter when predicting evolution. This is an unfortunate message, because virtually any application of evolutionary principles to real world systems will require many levels of similarly naive assumptions – implementation will never await knowing all details in advance (e.g., [23,24]).

Aside from the unexpected benefit of dispersin, several other observations reflect on our ability to predict or anticipate behavior of the transgenic phage: 

1 The transgenic phage dramatically augmented clearing of short-term biofilms in two different media but had only a modest effect in a third. Adding enzyme alone to short term biofilms could reproduce the effect. The effect of media was not explored further but could be a consequence of varying concentrations of PNAG production relative to other biofilm exopolysaccharides. These observations are broadly compatible with those expected from previous work [4] except for the effect of media.

2 The dispersin transgene offered no benefit in clearing long-term biofilms, the environment of greatest potential utility. The contrast with dispersin’s effect in short term biofilms was partly attributable to a different architecture of the two types of biofilms, suggesting that even preliminary laboratory studies on biofilm remediation should be focused on the conditions of end use.

3 During growth in both long- and short-term biofilms, the transgene was beneficial and its frequency increased at least temporarily. Whereas the evolutionary theory dictated that this benefit should stem from or be enhanced by spatial architecture of the biofilm, the benefit merely reflected the specific physiology of cells growing in a biofilm. Bacteria sloughed from the biofilms provided an equal or superior benefit to the transgenic phage. There was thus no insight or outcome attributable to the tragedy of commons framework.

The results indicate that maintenance of a transgene depends both on intrinsic properties of the transgene and on engineering. Although the tragedy framework did not obviously apply to dispersin maintenance, the basis of that model failure is a good one from an engineering perspective: the transgene was intrinsically beneficial. In cases where the transgene is intrinsically costly and a tragedy of the commons is not averted, nature may suggest ways to engineer a solution. Specifically, some naturally-occurring phage depolymerases enhance biofilm degradation [3,29,30]. Those enzymes are typically encoded as virion tailspikes and are required for infection, thus directly benefitting the parent genome; they are not subject to loss through a tragedy of the commons. Engineering enzymes as phage tail components may thus enable maintenance of a transgene when it would otherwise be lost.

## Methods

### Strains and media

Phage and bacterial strains are described in Tables 3 and 4. All are from the collections of JJB or IJM. LB broth was 10 g NaCl, 10 g Bacto tryptone, 5 g Bacto yeast extract per liter. $\frac{1}{3}$ LB used a third of all ingredients per liter. M9 glucose was 47.8 mM Na _2_*HPO*_4_, 22 mM KH _2_*PO*_4_, 8.5 mM NaCl, 1.87 mM NH _4_Cl, 1 mM MgSO _4_, 0.1 mM CaCl _2_ with 0.2% glucose per liter. Plates used LB with 1.5% Bacto agar. Determinations of phage titers used plates overlaid with soft agar (0.7% Bacto agar in LB) containing a suitable density of hosts.

**Table 4:** Bacterial strains

**Notation**	**Genotype:**	**Use**	**Ref**
IJ1133	*E. coli* K-12 F ^-^*ΔlacX74 thi Δ(mcrC-mrr)102*::Tn10	Host for measuring phage titers and some serial transfers to determine the evolutionary stability of the *dsp B* transgene in the absence of an extracellular PNAG matrix.	[31]
BL21 (DE3)	*E. coli* B Gal ^-^*dcm ompT hsdS*(r _*B*_- m _*B*_-) [ *λ*(DE3)]	Host for protein expression	[32]
IJ512	*E. coli* K12 *ΔlacX74 supE44 galK2 galT22 mcrA rfbD1 mcrB1 hsdS3* /F ^′^*lac*	Host for selecting T7 carrying T3 gene *1.2*	
*ΔtrxA*	JW5856 (Keio *ΔtrxA::kan*)	Host for selection of positive phage recombinants carrying *trxA*	KEIO [33]
*ΔcsrA*	*E. coli* K-12 F ^-^*ΔlacU169 ΔcsrA::kan*	Biofilm host; overproduces PNAG, the substrate for dispersin B	TR1-5 mutant in [34]
*ΔnagZ*	JW1083 (Keio *ΔnagZ::kan*)	Host with low background activity for producing dispersin B protein from phage lysates	KEIO [33]

The *E. coli trx*A gene, or alternatively T3 *1.2* was used as a selectable marker in detecting initial recombinants but has no benefit afterward in our design. Consequently, the evolutionary fate of the dispersin B gene may be considered to have been influenced by *trx*A. However, some adaptations used a recombinant mix that deleted *trx*A from the dispersin transgene, and other adaptations were observed to spontaneously delete *trx*A while maintaining dispersin. Thus *trx*A is not expected to affect the evolutionary fate of dispersin. All adaptations of phages in which T3 *1.2* had been used as a selectable marker were recombinant mixtures that included phages carrying dispersin but lacking T3 *1.2*, so again, this marker would not affect selection of the dispersin B gene.

### Plasmids

Plasmid pET15b-dspB was the commercial vector pET15b with an insert of bases 1-1086 of the dispersin B (dspB) ORF from *Aggregatibacter actinomycetemcomitans* CU1000 (Genbank AY228551). Although this fragment is not the complete ORF, it has beta-hexosaminidase activity [35]. pUC57-T7dspB consisted of the vector pUC57 with a linear insert in order of (i) T7Select 415b-1 bases 21403-21503 (Novagen), (ii) the *ϕ10* T7 promoter, (iii) a ribosomal binding sequence, (iv) bases 1-1086 of the *A. actinomycetemcomitans* dspB ORF, and (v) T7Select 415b-1 nts. 21517-21617. pUC57-T7trxAdspB consisted of pUC57-T7dspB with the *E. coli* trxA gene inserted between T7Select 415b-1 nts. 21403-21503 and the *ϕ10* promoter. pMKRQ-T71.2+dspB consisted of plasmid pMK-RQ (Gene Art) with an insert of T7 bases 11059-11229 (5’ of gene *3.8*) followed by T3 gene *1.2*, dspB, and T7 bases 11550-11652 (the 3’ end of *3.8*). pMKRQ-T7dspB was pMKRQ-T71.2+dspB without the T3 *1.2* gene.

### Recombinant phage construction

Recombinant phages were obtained by first plating T7v on a host containing a plasmid that carried an insert of dspB with a selectable marker (*trx*A or T3 *1.2*) and T7 flanking sequences. Plaques, which invariably contained a mixture of recombinants and T7v, were suspended and replated on an appropriate selection host. These second-step plaques were then usually purified in a subsequent round of plating and the presence of dispersin checked phenotypically and/or by PCR.

The phages at this step carried not only the dispersin gene but a selectable marker that might have imposed a fitness cost. To eliminate the selectable marker, phages were plated on a host carrying a plasmid that would recombine out the selectable marker but leave intact the dsp gene. In some cases, isolates lacking the selectable marker were identified by screening for inability to grow on the *Δ*trxA host and then confirmed by PCR. In other cases, the entire polymorphic plaque was used, as selection would be able to separate the selectable marker from dsp.

### Dispersin B protein expression and activity assay

BL21 (DE3) cells with pET15b or pET15b-dsp were grown to log phase in 10 mL of LB broth at 37°C. IPTG was added to 1 mM and the cultures grown for 3 hr. Cells were pelleted, resuspended in 0.5 mL of lysis buffer (100 mM NaCl, 20 mM Tris-HCl pH 8.0, 6 mM MgSO _4_ and 0.5 mg/mL lysozyme), subjected to two rounds of freeze-thaw and cleared by centrifugation. Lysates were stored at 4°C, maintaining activity for at least one month.

Chemical activity of dspB protein was assessed using the colorimetric substrate 4-nitrophenyl N-acetyl- *β*-glucosaminide (Sigma-Aldrich, St. Louis, MO) at 1 mM [17]. Commercial *β*-N-acetylglucosaminidase (New England Biolabs, Ipswitch, MA), an exoglycosidase, was used as an enzyme standard. Reactions followed manufacturer instructions using the recommended buffer. Reactions were incubated for 30 min at 37°C and stopped with 100 mM glycine pH 10. Absorbance at 405 nm was measured using the UV-Vis module of the NanoDrop Spectrophotometer (Thermo Scientific, Wilmington, DE).

One preparation of dspB enzyme used lysates of dispersin phages. *ΔnagZ* cells were grown at 37°C with aeration in 50 mL LB broth to a density of approximately 10^8^ cells/mL. 1×10^4^ phage per mL were added to each culture and the cultures grown until visible lysis (2 hr). Protein from lysates was precipitated with saturated ammonium sulfate, pelleted and resuspended in 0.5 mL of buffer (20 mM Tris-HCl pH 8, 50 mM NaCl, 1 mM EDTA, pH 8). For controls, phage-free lysates of *ΔnagZ* cells were grown, pelleted and lysed by freeze-thaw as described above. Protein from the cleared cell lysate was precipitated using saturated ammonium sulfate, pelleted and resuspended in 0.05 mL buffer.

### Short-term biofilms

Aliquots (100 *μ*L) of *ΔcsrA* cells were made as 10-fold concentrates of a stationary phase culture, frozen in LB with 20% glycerol. An aliquot was suspended in 12 mL media and 1 mL was added to each well of a 24-well tissue culture plate (Corning with Standard Tissue Culture Treated Surface). Cells were grown overnight in a static 37°C incubator. After 12–16 hr, the media was replaced and supplemented with 10^4^ – 10^5^ phage per well or with a lysate containing enzyme. The plate was then incubated 5 hr in a static 37°C incubator. After treatment, wells were rinsed once with water, air dried, stained for 15 min with crystal violet (CV, 0.1% in water), rinsed twice in water, and then air dried. Residual CV was extracted in 95% ethanol and quantified by absorbance at 560 nm using the UV-Vis module of the NanoDrop Spectrophotometer (Thermo Scientific, Wilmington, DE).

### Long-term biofilms

Biofilms of *ΔcsrA* cells were grown in $\frac{1}{3}$ LB for 7 days in silicone tubing (20 cm, 0.125" I.D. x 0.250" O.D; VWR International LLC, Radnor, PA). An additional length of tubing connected the biofilm chamber to a waste container. Luer-lock stopcocks at each end of the chamber tubing allowed sterile introduction and sampling of phage and cells. Media flow rate was kept at 3 mL/hr. The biofilm chambers were placed on a covered slide warmer set at 37°C. The cell preparation was the same as for short-term biofilms. After inoculation, cells were incubated statically for 1 hr to allow attachment prior to reinstating media flow.

Biofilms were grown for 7 days, then 10^4^ phage were added. The biofilm was grown a further 4–5 days. Biofilm effluents were assayed for phage daily. After phage treatment, chambers were rinsed once with water and dried. Crystal violet staining assessed biofilm mass.

### Growth of phage for testing loss of transgene

Evolutionary stability of transgene maintenance was carried out on IJ1133 in LB or on *ΔcsrA* in $\frac{1}{3}$ LB. Eight lines used 3 hr of serial transfer on IJ1133 following established methods [36,37]. One line was grown 48 hr by continuous culture in a 2-tube chemostat [38].

### Statistics of dispersin transgene frequency

Standard PCR was used to measure the presence or absence of the dispersin gene in phage isolates to obtain frequency estimates from a population. Primer pairs consisted of one internal and one flanking dispersin, or both flanking. When the frequency of the target sequence is high or low, nearly all phage will be of the same type, and a large number of single-plaque reactions are required to estimate the frequency. Combining multiple plaques into a single reaction facilitates the detection of uncommon types by reducing the number of reactions needed. However, the statistics of sampling error become somewhat more complicated. We used the following methods:

Suppose the population frequency of phages carrying a transgene is *T* (*U*=1-*T* for phages lacking the transgene). Let *n* phage isolates be combined into a single PCR reaction such that the product reveals either (A) the absence of the transgene in all *n* isolates (probability *U*^*n*^), (B) the presence of the transgene in all *n* isolates (probability *T*^*n*^), or (C) the presence of the transgene in some but not all isolates (probability 1-*U*^*n*^-*T*^*n*^). (Multiple primer sets of the same mixture of plaques may be required to distinguish all 3 outcomes.) If *R* independent *n*-plaque reactions are tested, the probability of observing *X* reactions in which all *n* isolates lack the transgene, *Y* reactions in which all isolates carry the transgene, and *Z* reactions in which at least one plaque of each type occurs is the corresponding multinomial term,

$$M(A,B,C,X,Y,Z)\;\propto \;A^{X}B^{Y}C^{Z}\;\;\;\;\;\;\;\;\;\;,$$

where *A*=*U*^*n*^, *B*=*T*^*n*^, and *C*=1-*U*^*n*^-*T*^*n*^ and *X*+*Y*+*Z*=*R* (the combinatorial constant term is omitted). *M*(*A*,*B*,*C*,*X*,*Y*,*Z*) is easily solved for the maximum likelihood estimate of *T*, and a 95% Bayesian ‘credible’ interval obtained by treating the normalized *M*(*A*,*B*,*C*,*X*,*Y*,*Z*) as a probability density function of *T* (0≤*T*≤1) and finding the bounds for the upper and lower 2.5% tails.

### Graphics

Figures were drawn in R [39].

### Simulations

Differential equations were evaluated numerically in the program Berkeley Madonna (v. 9.0.118 beta) with a step size of 10^-3^ and the Euler method. The numerical output was transferred to R for presentation.

## Competing interests

The authors declare that they have no competing interests.

## Authors’ contributions

MS developed many of the assays reported here and carried out most of them; NC carried out some fitness assays. IJM advised the conduct of several assays and the overall molecular biology dimension of the study and helped write the paper. DA and JT conducted some preliminary adaptations of Tim Lu’s T7 dispersin phage at the University of Idaho. JJB designed the study, oversaw its conduct, did the math, and wrote most of the paper. All authors read and approved the final manuscript.
